# Digital spatial profiling identifies features of primary and locoregional metastatic vasculature in triple negative breast cancer

**DOI:** 10.1007/s10585-026-10391-4

**Published:** 2026-01-19

**Authors:** Akhilandeshwari Ravichandran, Kyle Upton, Shiva Taheri, Cheng Liu, Kaltin Ferguson, Mark Adams, Laura J. Bray

**Affiliations:** 1 Centre for Biomedical Technologies, Queensland University of Technology (QUT), 60 Musk Ave., Kelvin Grove, QLD 4059 Australia; 2 School of Mechanical, Medical and Process Engineering, Faculty of Engineering, Queensland University of Technology (QUT), 2 George St, Brisbane City, QLD 4000 Australia; 3 Central Analytical Research Facility, Queensland University of Technology (QUT), 2 George St, Brisbane City, QLD 4000 Australia; 4 Pathology Queensland, Princess Alexandra Hospital, Brisbane, QLD 4102 Australia; 5 Faculty of Medicine, The University of Queensland, Herston, QLD 4006 Australia; 6 Mater Research Institute - The University of Queensland, Translational Research Institute, 37 Kent Street, Woolloongabba, QLD Australia; 7 Mater Health Services, South Brisbane, QLD 4101 Australia; 8 Faculty of Health, Centre for Genomics and Personalised Health, School of Biomedical Sciences, Queensland University of Technology (QUT), 60 Musk Ave., Kelvin Grove, QLD 4059 Australia; 9 ARC Training Centre for Cell and Tissue Engineering Technologies, Queensland University of Technology (QUT), 60 Musk Ave., Kelvin Grove, QLD 4059 Australia

**Keywords:** Spatial profiling, Microvasculature, Metastasis, TNBC

## Abstract

Triple Negative Breast Cancer (TNBC) is an aggressive subtype of breast cancer with limited treatment options and poor prognosis. Coupled with this, TNBC shows a high rate of metastasis which is known to be aided by tumour vasculature. Endothelial cells that form the lining of the tumour vasculature exhibit distinct genotype and phenotype differences compared with normal tissue vasculature. However, little is known about endothelial signatures that drive metastasis from a primary tumour, particularly in the context of immune infiltration. In this study, we utilized GeoMX Digital Spatial Profiling to investigate spatial proteomics differences in endothelial cells between primary and secondary sites of TNBC. By segmenting tissues using epithelial (PanCK), immune (CD45), and endothelial (CD31) markers, we analysed the microvasculature for a panel of 79 target proteins. In paired primary and secondary TNBC tissues, we identified significant downregulation of fibronectin (− log2(Fold-Change) = − 1.7, *p* < 0.001) in secondary sites. Specifically in epithelial regions, S100B was found to be downregulated in secondary microvasculature when compared to primary tumours. Additionally, metastasis-free primary tissues exhibited upregulated expression of S100B when compared to primary tissues that metastasized. Our study highlights the potential contributions of microvasculature to metastatic progression in TNBC, presenting new opportunities to explore them as potential biomarkers of TNBC metastasis.

**Supplementary Information:**

The online version contains supplementary material available at 10.1007/s10585-026-10391-4.

## Introduction

More than 2 million women are diagnosed with breast cancer globally each year and it remains one of the most common cancer types in women [1]. With advances in early detection and treatment, the 5-year survival rates have consistently risen over the past decade in most breast cancer subtypes including triple negative breast cancer (TNBC). TNBC is a breast cancer subtype marked by the absence of hormonal receptors—estrogen and progesterone, and Human epidermal growth factor receptor-2 (Her-2), making it difficult to target and treat. While immunotherapy has shown promising implications for TNBC [2], the survival rates associated with metastasized TNBC are still quite low with no standardized treatment available [3]. Consequently, it has been an active area of research with investigations into the mechanistic pathways involved in TNBC metastasis [4]. Especially, the role of angiogenesis and tumour vasculature in disease progression and metastasis has been previously studied [4–7]. This has prompted the investigation of several anti-angiogenic therapies over the years to block tumour vasculature and target growth of the tumour. However, there is limited evidence of their long-term clinical efficacy due to challenges including therapeutic resistance, relapse, and lack of validated biomarkers [8].

Tumour vasculature has been shown to have an abnormal phenotype that is reflected in its morphology, barrier capabilities and permeability [9–11]. Previous studies have identified factors linked to this abnormality in an effort to normalize it and prevent leaky blood vessels [12–14]. In addition to differences between normal and tumour vasculature, studies have shown heterogeneity in vessel morphology, formation and function of tumour vasculature even between primary and locoregional secondary sites [15–17]. For instance, anti-angiogenic therapies were shown to have no effect on lymph node metastases highlighting that the secondary site vasculature signatures differ from those of primary tumours [17]. While TNBC is known for its high incidence of lymphatic metastasis, the role of microvasculature in its metastatic progression remains unclear. Also, little is known regarding the phenotypic heterogeneity of the primary tumour-associated vasculature when compared to matched metastatic sites. Extracting this information is met with the practical challenge associated with isolating microvasculature from primary and secondary tumour sites. With endothelial cells comprising only 2.5% of viable cells that can be isolated from TNBC tissue, conducting in-depth single-cell analyses of the tumor microvasculature presents a significant limitation [18]. To address this knowledge gap, we have used spatial proteomic assays to map the changes in signalling pathways of endothelial cells localised in TNBC tissues and locoregional metastatic tissues.

## Results

### Patient characteristics

To investigate the role of microvasculature in TNBC metastasis, we prepared two tissue microarrays (TMAs) from archival samples of primary and metastatic tissue samples from women with TNBC. These included 21 treatment-naïve samples and 2 metastatic samples (paired, collected post-chemotherapy) sourced from Mater pathology. Table 1 outlines clinical pathological details of the patient tissue samples in the TMAs, along with the information about the selected regions of interest (ROIs). Of the samples, 11 had paired primary and metastatic sites (11 nodal metastases and 1 chest wall metastasis). The TMA also incorporated metastasis-free primary-only samples to compare primary sites that did metastasise (*N* = 11) with those that do not (*N* = 10) as well as 1 metastasis-only sample. Hematoxylin and Eosin (H&E) staining was done on the slides to help annotate the slides for ROI selection prior to spatial profiling (Supp Fig. 1).

**Table 1:** Clinicopathological details of patients in the study

	Primary	Metastases
Paired metastatic		
N	11	11 (node), 1 (chest wall)
No of ROIs	31	35
Median age (range)	62 (43, 88)	
Deceased	4	
Grade	3 (9), 2 (2)	3, N/A
Metastasis-free		
N	10	−
No of ROIs	25	
Median age	61 (41, 86)	
Deceased	−	
Grade	3 (8), 2 (2)	
Metastatic (not-paired)		
N	1	1
No of ROIs	2	3
Median age	76 (52, 86)	
Deceased	−	
Grade	3	3

### Defining microvascular, epithelial, and immune landscapes of primary and secondary TNBC sites

**Fig. 1:**
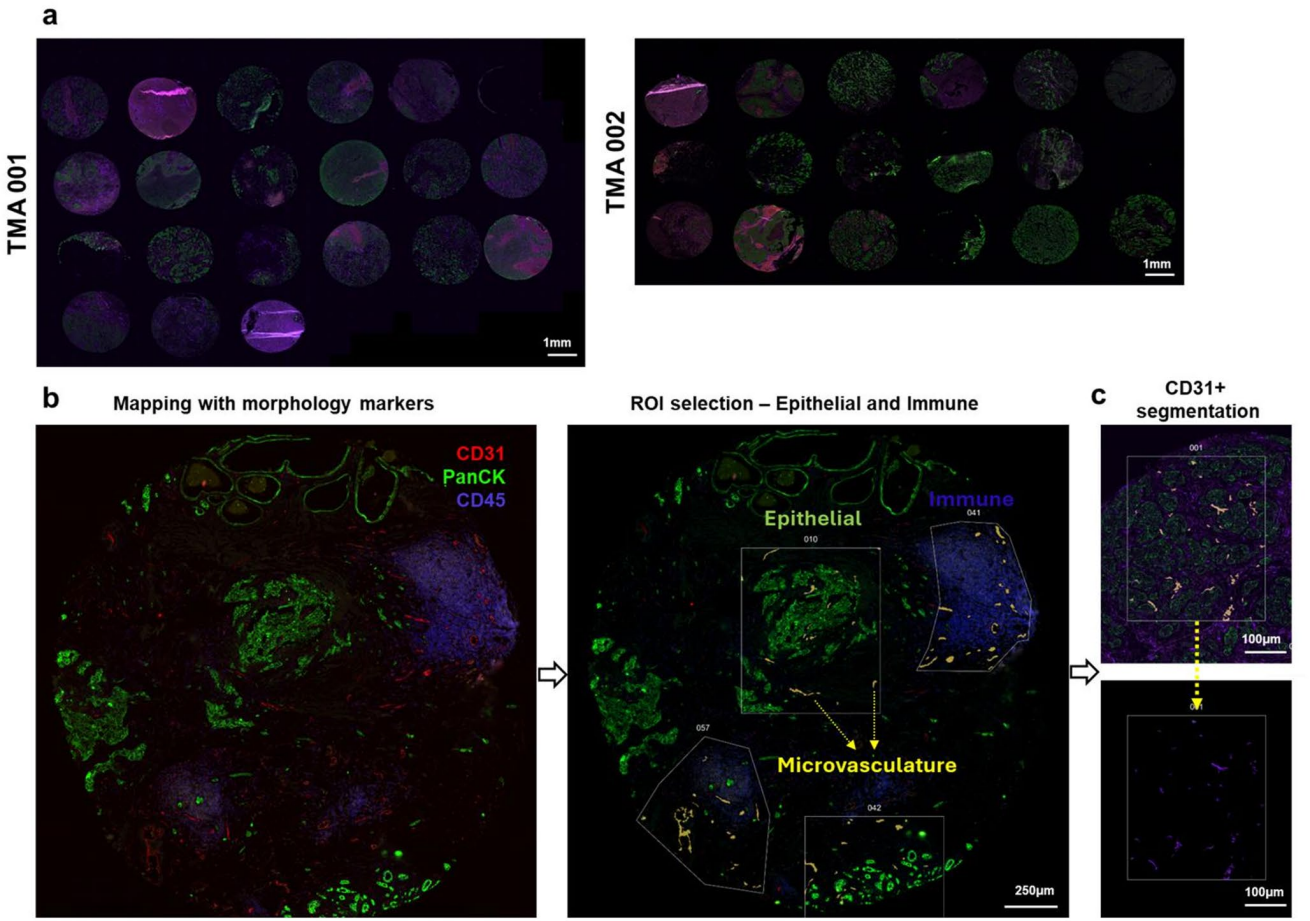
Region of interest (ROI) selection and CD31^+^ cell-based segmentation using GeoMx DSP. **a** Overview of TMAs with TNBC patient tissue sections (both primary and metastatic) used in this study (Scale bar (white): 1 mm). **b** Representative image of a core stained for CD31^+^  microvasculature (red), PanCK^+^epithelial regions (green), CD45+ immune-infiltrated regions (purple). The selection of regions of Interest (ROIs) is shown to distinguish microvasculature in epithelial versus immune-infiltrated regions (Scale bar (white): 250 μm). **c** Cell-type specific segmentation of CD31^+^ cells within a representative ROI (Scale bar (white): 100 μm)

The TMAs were stained to map the TNBC patient tissues sections using morphology markers to identify microvasculature (CD31^+^), epithelial regions (PanCK^+^), and immune-infiltrated regions (CD45^+^) (Fig. 1a, b). Using digital spatial profiling, regions of interest (ROIs) were selected to assess differentially expressed proteins in the CD31 + microvasculature, with segmentation between the epithelial and immune-infiltrated regions (Fig. 1c).

**Fig. 2:**
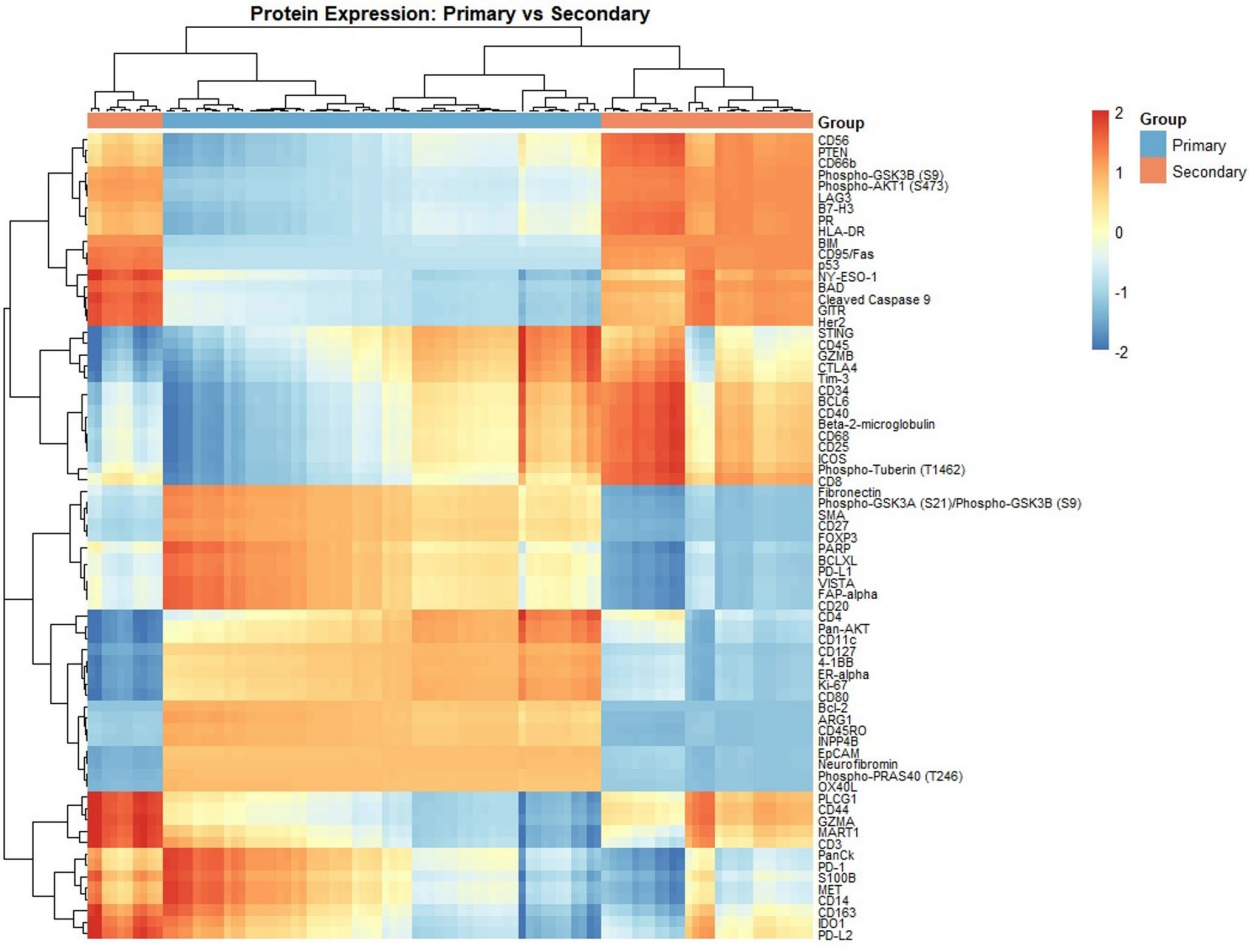
Heatmap representing broad classification of differential expression. Overall heatmap following hierarchical clustering to visualize differential protein expression between primary and secondary microvasculature

**Fig. 3:**
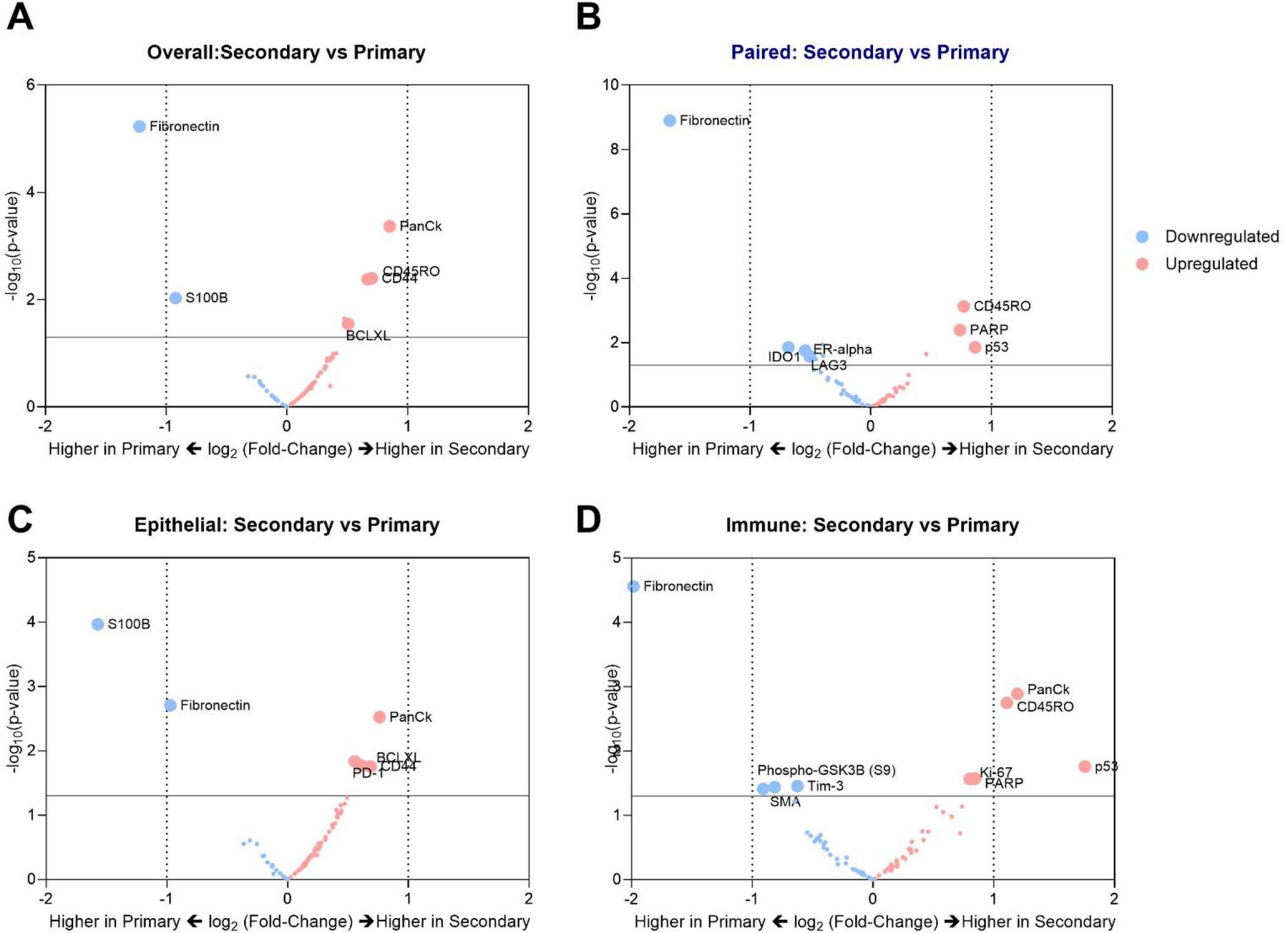
Microvascular signatures of secondary versus primary TNBCs. Volcano plots of **a** Differential expression of microvasculature proteins in overall secondary TNBC patient tissues (*N* = 12) versus primary TNBC patient tissues (*N* = 22). **b** Differential expression analysis of microvasculature in paired secondary and primary sites of TNBC patient tissues (*N* = 11). **c** Differential expression of microvasculature in the epithelial regions (PanCK^+^ areas) and **d** Immune-infiltrated regions (CD45^+^ areas) of secondary versus primary TNBC patient tissues. Upregulation and downregulation of proteins in secondary sites when compared to primary sites are marked in red and blue respectively. Dotted vertical lines represent log_2_(Fold-Change) of − 1 and 1. Proteins above the solid horizontal line at − log_10_(p-value) = 1.3 marks *p* < 0.05

First, we conducted an overall comparison of CD31^+^microvasculature in secondary sites (*N* = 12) and primary TNBC sites (*N* = 22) using differential expression (DE) analyses (Fig. 2). The results revealed downregulation of fibronectin in secondary sites (Log_2_(Fold-Change) = − 1.2, *p* < 0.001) (Fig. 3a). Next, we performed DE analyses on a smaller subset of 11 patients with paired primary and secondary tissues to identify differences in microvascular expression between primary tumours and their paired secondary sites. In the paired analysis, fibronectin was again found to be significantly downregulated (Log_2_(Fold-Change) = − 1.7, *p* < 0.001) in the secondary sites (Fig. 3b).

To examine region-specific differences in protein expression within microvascular structures at secondary and primary sites, CD31^+^ cells in epithelial regions (PanCK^+^) and immune-infiltrated regions (CD45^+^) were compared across both sites in TNBC patients. Secondary sites exhibited a downregulation of S100B specifically in the epithelial regions (Fig. 3c). In the immune-infiltrated regions, secondary sites showed an upregulation of PanCK, CD45RO and p53, along with a downregulation of fibronectin compared to primary sites (Fig. 3d). Further, differences were observed between epithelial and immune-infiltrated regions within primary and secondary sites. In primary sites, CD31^+^ cells in epithelial regions exhibited an upregulation of PanCK, S100B and p53 compared to immune-infiltrated regions (Supp Fig. 2a). In contrast, epithelial regions in secondary sites showed an upregulated of fibronectin and downregulation of CD45 compared to immune regions (Supp Fig. 2b).

### Impact of size of immune infiltration on microvascular protein expression

**Fig. 4:**
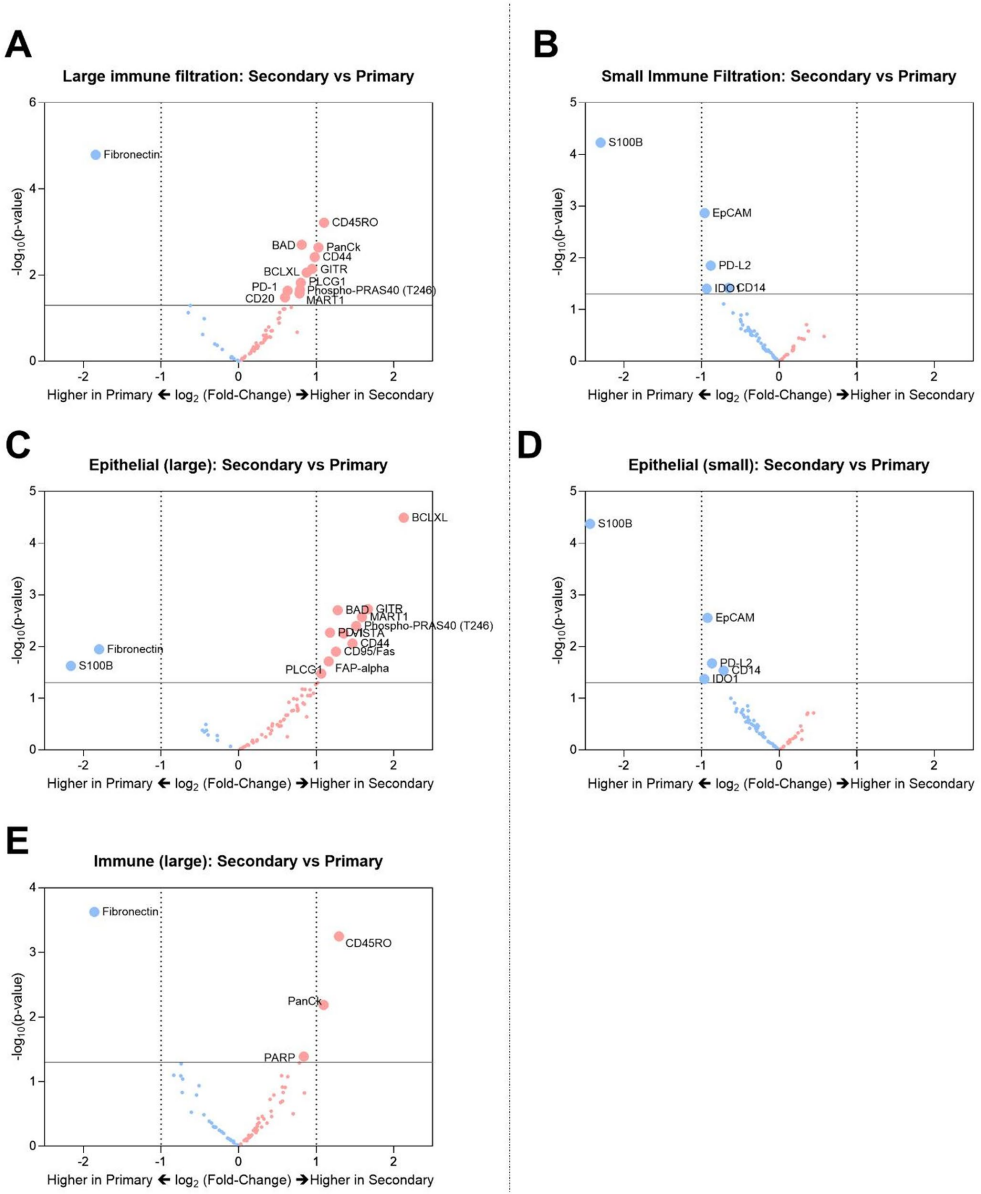
Influence of size of immune infiltration on microvascular signatures of secondary versus primary TNBCs. Volcano plots of differential expression of microvascular proteins in secondary versus primary TNBCs in **a** Large immune infiltrated sites, **b** Small immune infiltrated sites. Volcano plots of differential expression of microvascular proteins in secondary versus primary sites within epithelial regions with **c** Large immune infiltration and **d** Small immune infiltration and **e** Immune-only regions

Immune infiltration in TNBC tissues is associated with an improved prognosis for patients [19, 20]. Therefore, we aimed to explore the impact of size of immune infiltration on endothelial cell protein expression in both primary and secondary TNBC sites. ROIs were classified as ‘large immune infiltration’ when the ROI was predominantly CD45^+^ rich regions with little or no epithelial cells. Smaller immune infiltration ROIs were characterized by sparse distribution of CD45^+^ immune cells. In primary tissues, large immune infiltration was associated with lower expression of p53, PanCK, and S100B (Supp Fig. 3a). In contrast, large immune infiltration in secondary sites was associated with upregulation of CD44, CD45, and OX40L and downregulation of p53 compared to small immune infiltration (Supp Fig. 3b). Drawing out differences between primary and secondary sites based on immune infiltration size, we observed a downregulation of fibronectin in secondary sites with large immune infiltration when compared to primary sites (Fig. 4a). And smaller immune infiltration in secondary sites was associated with downregulation of S100B (Fig. 4b).

Next, we wanted to understand the influence of size of immune infiltration on the microvascular expression within epithelial and immune landscapes. Here, epithelial regions in secondary sites with larger immune infiltration showed downregulated expression of S100B and fibronectin, and an upregulation of BCLXL, PD-1, PD-L1, BAD, GITR, MART-1, FAP-alpha, etc. (Fig. 4c). With smaller immune infiltration, the epithelial regions in secondary sites showed downregulated expression of S100B (Fig. 4d). Irrespective of size of immune infiltration, the epithelial regions were marked by downregulation of S100B in secondary sites when compared to primary sites. Larger immune infiltration specifically showed a downregulation of fibronectin in the epithelial secondary sites when compared to primary. Similarly, immune landscapes showed downregulation of fibronectin in secondary sites when compared to primary sites (Fig. 4e).

### Comparison of microvascular expression in metastatic versus metastasis-free primaries

**Fig. 5:**
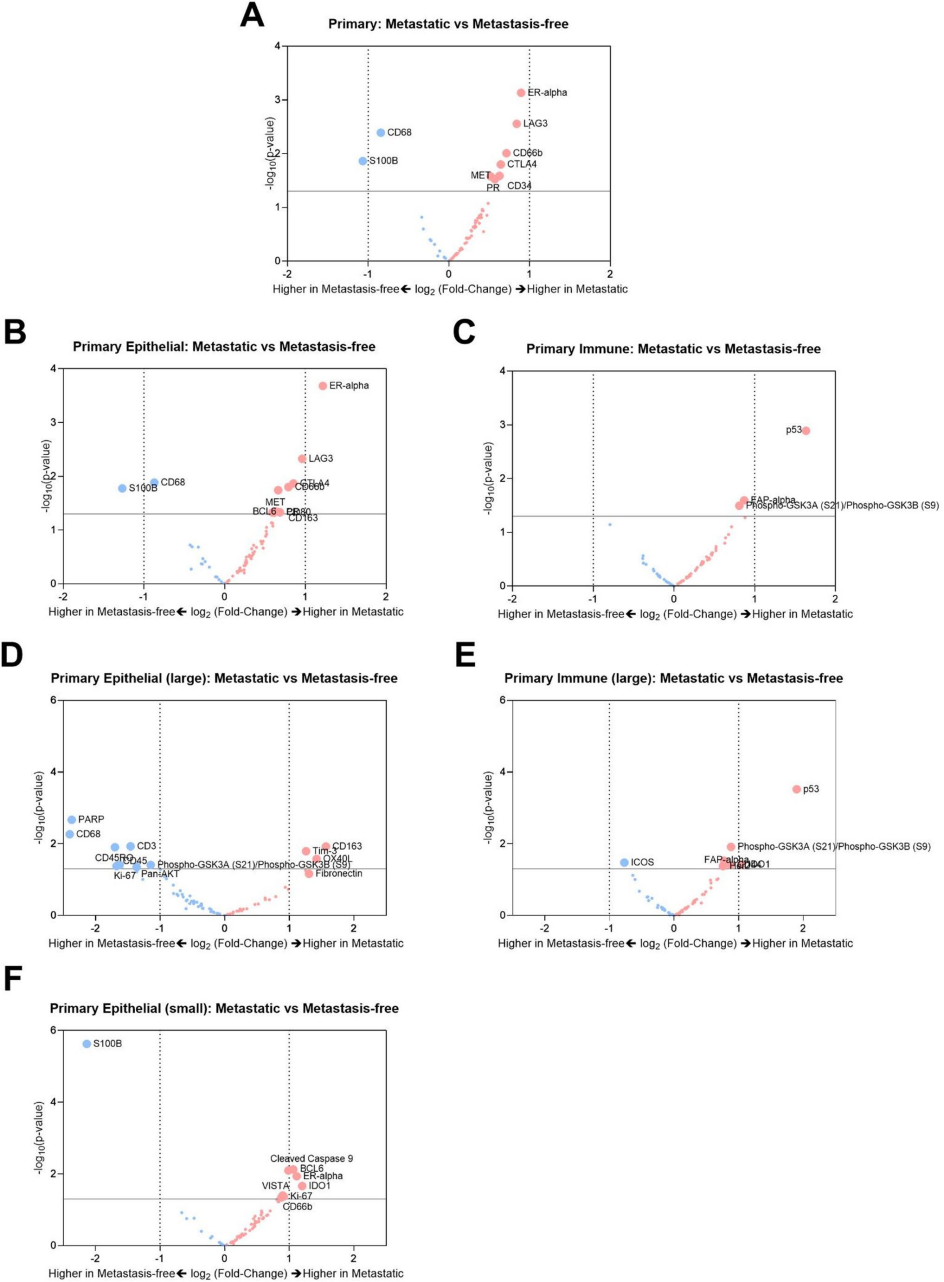
Stratification of microvascular signatures of metastatic versus metastasis-free primary TNBCs.** a** Volcano plot of differential expression of microvascular proteins in metastatic versus metastasis-free primary TNBCs. Volcano plots of differential expression in of microvascular proteins in metastatic versus metastasis-free primary TNBCs within **b** epithelial regions, **c** immune-infiltrated regions, **d** epithelial regions with large immune infiltration, **e** Large immune infiltrated sites, and **f** Epithelial regions with small immune infiltrated sites

Next, we wanted to identify distinguishing features of microvasculature in primary TNBC sites that did not metastasize compared with those that did. Differential expression analyses showed that TNBC microvasculature had downregulated expression of S100B in metastatic primary sites when compared to metastasis-free sites (Fig. 5a). And this trend was reflected only in the epithelial sites (Fig. 5b) and not observed in the immune infiltrated regions (Fig. 5c). Microvasculature in the epithelial regions in metastatic primary sites had shown upregulated ER-alpha, with large immune infiltrated sites showing upregulation of CD163, Tim-3, OX40L and downregulation of PARP, CD68, CD3, CD45RO and CD45 (Fig. 5d). Microvasculature in the immune-only regions of metastatic primary sites showed an upregulation of p53 and IDO-1 (Fig. 5e). Interestingly, epithelial regions with small immune infiltration showed downregulation of S100B in ECs in primary sites of metastatic TNBC (Fig. 5f).

## Discussion

Accounting for 20% of women with breast cancer, TNBC is an aggressive cancer that has a higher frequency of metastases and poor prognosis compared to other types of breast cancer [21]. In the absence of regional or distance metastases, localized TNBC has better prognosis and survival outcomes with the 5-year survival rate dramatically reducing from 91% to 66% in TNBC patients with regional metastases [3]. Previous research has shown that vasculature and angiogenic factors are important contributors to this metastatic progression in TNBC and other cancer types [22]. This prompted the development of anti-angiogenic therapies that have been studied for more than half a century with almost all of the clinically approved drugs targeting the VEGF pathway [23]. By blocking the VEGF pathway, the drugs have been shown to either directly disrupt angiogenesis or normalize tumour vasculature to improve drug delivery or immune responses [24]. While they have shown highly promising results preclinically, these anti-angiogenic therapies have resulted in limited clinical outcomes because of resistance to drugs and has often warranted combination with other therapeutic modalities [24]. Inhibition of angiogenesis has been shown to activate compensatory pro-angiogenic mechanisms and pathways that protect the vascular supply to the tumours [25]. Another important challenge associated with antiangiogenic therapies is the lack of reliable biomarkers for predicting therapeutic response. This necessitates better characterization of tumour vasculature to identify and validate potential biomarkers to support the development of novel therapeutic combinations targeting the angiogenic pathways.

By investigating vasculature in primary tumours and metastasized tumours, we can understand the angiogenic mechanisms behind disease progression and evolution, thereby enabling the development of strategies to target and prevent metastasis. We had previously demonstrated the application of spatial transcriptomics to identify whole-transcriptome-wide differences of microvasculature obtained from tumour sites and normal adjacent sites of TNBC patients [12]. In this study, we have compared the microvasculature of primary and secondary sites of TNBC within epithelial-rich regions and immune-rich regions using spatial proteomics.

Several studies have previously done a deep dive into molecular mechanisms that lead to development of metastatic breast cancers [26, 27], with some focussed on differences observed in specific cell types such as immune cell [28] and endothelial cells [29]. In a comparison of matched primary and distant metastatic HER2^+^ breast cancers (soft tissue, lung, brain), Schlam et al. showed that primary sites had higher tumour infiltration, increased expression of immune activation markers compared to secondary sites [27]. In a similar study although with unmatched primary and metastatic lesions, Rozenblit et al. showed the differential expression of Programmed Death Ligand 1 (PD-L1) expression in primary tumours and different metastatic sites. Interestingly, they found pronounced differences between primary tumours and distant metastases such as liver, skin and bone when compared to nodal metastases [28]. Only a small number of studies have highlighted differences between primary tumours and regional/nodal metastases to determine factors that mediate early dissemination and disease progression [30–32]. For example, single-cell RNA sequencing (scRNA-seq) and spatial transcriptomics have revealed a metabolic shift in tumour cells in primary invasive breast tumours and paired metastatic axillary lymph nodes [31]. However, it is important to note that most of the above studies are not specific to microvasculature within the primary and the secondary sites of metastasis. Very few studies have correlated endothelial signatures involved in the progression of metastatic breast cancers [29, 33]. Considering the contribution of vasculature in disease progression, profiling the endothelial cells in TNBC would provide valuable insights and potential biomarkers of TNBC prognosis.

In our study, overall and paired comparisons of primary and secondary TNBC microvasculature revealed downregulated expression of fibronectin in secondary TNBC sites, especially associated with immune-infiltrated regions. Fibronectin is a matrix protein that is crucial for cellular adhesion and migration, with contradicting and controversial reports of its role in metastatic progression [34–38]. However, there is little research on the significance of fibronectin expression in tumour microvasculature, especially in the context of metastasis [39, 40]. Increased fibronectin deposits, especially associated with tumour vasculature, has been implicated in promoting vessel barrier dysfunction and tumour extravasation. Interestingly, fibronectin did not seem to stand out in the comparison between metastatic and metastasis-free primary microvasculature in our study. Given the role of stromal components in fibronectin secretion, we investigated the presence of fibroblasts in these tissues (alpha Smooth Muscle Actin [aSMA]). Our results did not show substantial differences between primary and secondary tissues (Supp Fig. 4). This highlights the importance of our study in defining differential protein expression within specific cell types (endothelial cells) in the context of the tissue section. Another prominent differentially expressed protein in this study was S100B, that belongs to a family of proteins that are ligands to the receptor family called RAGE (Receptor for Advanced Glycation End-products) [41]. Largely, S100B proteins have been studied with regards to neural disorders owing to their presence as a secreted molecule and a biomarker of distress in the brain [42]. However, studies have highlighted the role of RAGE in the metastatic progression of several cancers [43–45]. Not only did we observe decreased expression of S100B in secondary sites, but we also observed higher expression of S100B in primary microvasculature that were metastasis-free when compared to the metastatic primary microvasculature. These results confirm with Yen et al. who had reported better prognosis and metastases-free survival in breast cancer patients with high S100B expression, reiterating their potential role as a biomarker for TNBC prognosis [46]. Additionally, S100B has been reported to play a role in the development and sustaining of pro-inflammatory microenvironment. In microvasculature, S100B treatment has resulted in NF-kB activation and upregulation of vascular cell adhesion molecule 1 (VCAM-1) [47] and monocyte chemoattractant protein 1 (MCP-1) [48]. S100B has also been implicated as an intracellular regulator and is known to activate endothelial cells by elevating leukocyte adhesion to endothelial cells [49]. These reports suggest a link between S100B expression in microvasculature and immune cell recruitment. Further research may be required to understand the relationship between higher S100B expression in TNBC primary and sustenance of an inflammatory microenvironment that could render them metastasis-free. Additionally, epithelial microvasculature showed upregulated ERa expression in metastatic primaries when compared to metastases-free primary patients. This may be because our study had patient samples scored ER-ve (and hence TNBC) where the ERa staining was < 1%. Another recent protein analysis of responsive and non-responsive TNBC patients to adjuvant chemotherapy also showed increased expression of ERa in tumour regions of TNBC patients [50].

Specifically in TNBC, it is critical to consider the influence of immune pathways in prognosis and disease progression. With higher immune cell infiltration in TNBC, immune checkpoint blockade has become a promising therapy to treat this aggressive form of breast cancer. Stratification of immune-cold and immunoreactive TNBC tumour microenvironments has been successfully shown to associate these subtypes with outcomes and targets [51]. Through RNA profiling, Szekely et al. reported an immune inert environment in metastatic sites when compared to primary sites by mechanisms including immune-cell depletion, immunosuppression and immune-evasion [52]. We observed an upregulation of IDO-1 in immune regions of metastatic primary microvasculature. Previous reports had identified IDO-1 to be associated with improved survival outcomes in TNBC [51, 53]. Similarly, our DE analyses showed higher expression of ER-alpha in metastatic primary sites when compared to metastasis-free counterparts. Stromal ER-alpha has been reported previously to increase angiogenesis and tumour development [54] and indicative of better overall survival in TNBC patients [55]. In our study, we have not linked signatures with outcomes or treatment responses and future research in this direction may add valuable insight into the obtained dataset [56, 57]. We would like to acknowledge that 2 out of the 23 tissues were obtained post neoadjuvant chemotherapy, and it is important to consider variations that may arise due to the potential impact of treatment. Our strategy for microvasculature selection in DSP has been CD31 + cells [12], but it must be noted that this marks both vascular and lymphatic endothelial cells. While lymphatic endothelium is primarily involved in nodal metastasis, vascular endothelium is largely known to be involved in systemic metastasis. By selecting endothelial cells using CD31, the dataset can be utilized in future studies involving distant metastases. By performing a D2-40 staining for specifically targeting lymphatic endothelial cells, we observed positive staining for D2-40 in less than ~ 40% of the tissue cores tested in the TMA (Supp Fig. 5), with no differences observed between primary and secondary cores. Also, with tumour progression, endothelial-to-mesenchymal transition (EndMT) is known to cause endothelial cells to lose CD31 [58]. While this phenomenon may not alter all cells, it would be also be practically challenging to perform spatial profiling while being cognizant of changing morphology markers.

In summary, the study has applied spatial proteomics techniques on clinical patient samples to identify differential expression of several proteins including S100B and fibronectin between primary and secondary vasculature of TNBC. Region-specific differences were also observed between the sites in epithelial and immune regions of the tissues. Further, in agreement with previous research findings, our study demonstrated that lower S100B expression in primary TNBC tissue can be used as a prognostic marker of increased metastatic potential. Future studies will aim to exploit these protein signatures to normalize tumour microvasculature and prevent metastatic progression.

## Methods

### Patient-derived tissue sections on TMA

In this study we used archival tissues obtained from triple negative breast cancer patients undergoing treatment at Mater Hospital. Study was conducted in accordance with ethics approval from the Mater Misericordiae Ltd and Queensland University of Technology Human Research Ethics Committees (Approval numbers: HREC/17/MHS/50; 7472). Two tissue microarrays of primary and secondary TNBC patient tissues were prepared with core sizes of 2 mm.

### Nanostring DSP and protein profiling

The TMA slides were analyzed using the GeoMx^®^ Digital Spatial Profiling platform at Queensland University of Technology (QUT) by Spatial Genomics at QUT Central Analytical Research Facility. The TMA slide was prepared according to the Manual Protein Slide Preparation Protocol outlined in the GeoMx DSP Slide Preparation user Manual (Nanostring MAN-10138-06). The slide was probed with a cocktail of 86 antibodies from several panels including the human immune cell profiling core panel (NSTG121300101; v1.1), immuno-oncology drug target (NSTG121300102; v1.1), immune activation status (NSTG121300103; v1.0), immune cell typing (NSTG121300104; v1.0), pan-tumour (NSTG121300105; v1.0), cell death (NSTG121300112; v1.2), and PI3K/AKT panels (STG121300113; v1.0), along with antibodies for negative IgGs and housekeeping proteins. These antibodies were conjugated to unique UV-Photocleavable oligonucleotide tags. To distinguish various morphology, epithelial cells were labelled with PanCK, immune cells were labelled with CD45, endothelial cells were labelled with CD31, and nuclei were stained with SYTO13. Overall, 96 ROIs were assessed, with 58 ROIs in TMA 001 and 38 ROIs in TMA 002.

### Bioinformatics data analysis

For the NanoString DSP protein data, data quality was initially assessed using default settings in the DSP analysis suite. Data was then further validated and processed through a pipeline available at github.com/kyleupton/DSP_EDA_Protein. Briefly, systematic errors were checked, and a limit of detection (LOD) threshold was chosen. Data was first normalised to ERCC control probes then to “house-keeping” probes. Differential expression analysis was conducted in EdgeR using a GLM approach that accounted for sample factors such as tumour site (primary/secondary), tissue type (epithelial/immune), and size of immune infiltration [59]. *P*-Values were adjusted for multiple comparisons using Benjamini–Hochberg. EdgeR results were visualised using Matplotib.

### Statistical analysis

All results are expressed as mean ± standard deviation (SD). Data analysis and visualisations were performed using GraphPad Prism 10.0.1. Violin plots were analysed using an ordinary one-way ANOVA with multiple comparisons. A *p* value of < 0.05 was considered as statistically significant, and results are represented as *=p < 0.05, **= p < 0.01, ***= *p* < 0.005, ****= *p* < 0.0001.

## Supplementary Information

Below is the link to the electronic supplementary material.

Supplementary Material 1

## Data Availability

The data that support the findings of this study are available upon request.
