# Supplementary material for: Digital spatial profiling identifies features of primary and locoregional metastatic vasculature in triple negative breast cancer
Source: Clin Exp Metastasis. 2026 Jan 19;43(1):11. doi: 10.1007/s10585-026-10391-4 (PMC12812774; doi:10.1007/s10585-026-10391-4)
Supplement: Supplementary file 1 — Supplementary Material 1 [file 10585_2026_10391_MOESM1_ESM.docx]

Supplementary Figures

Defining biomarkers of primary and metastatic vasculature of Triple Negative Breast Cancer

**Akhilandeshwari Ravichandran^1,2^, Kyle Upton^3^, Shiva Taheri^1,2^, Cheng Liu^4,5^, Kaltin Ferguson^6,7^, Mark Adams^8,9^, Laura J Bray^1,2,9,*^**

^1^Centre for Biomedical Technologies, Queensland University of Technology (QUT), 60 Musk Ave., Kelvin Grove, QLD 4059, Australia.

^2^School of Mechanical, Medical and Process Engineering, Faculty of Engineering, Queensland University of Technology (QUT), 2 George St, Brisbane City QLD 4000, Australia.

^3^Central Analytical Research Facility, Queensland University of Technology (QUT), 2 George St, Brisbane City QLD 4000, Australia.

^4^Pathology Queensland, Princess Alexandra Hospital Brisbane, QLD 4102, Australia

^5^Faculty of Medicine, The University of Queensland, Herson, QLD 4006, Australia

^6^Mater Research Institute - The University of Queensland, Translational Research Institute, 37 Kent Street, Woolloongabba, QLD, Australia.

^7^Mater Health Services, South Brisbane, QLD 4101, Australia

^8^Centre for Genomics and Personalised Health, School of Biomedical Sciences, Faculty of Health, Queensland University of Technology (QUT), 60 Musk Ave., Kelvin Grove, QLD 4059 Australia

^9^ARC Training Centre for Cell and Tissue Engineering Technologies, Queensland University of Technology (QUT), 60 Musk Ave., Kelvin Grove, QLD 4059 Australia.

*Corresponding author, email: laura.bray@qut.edu.au


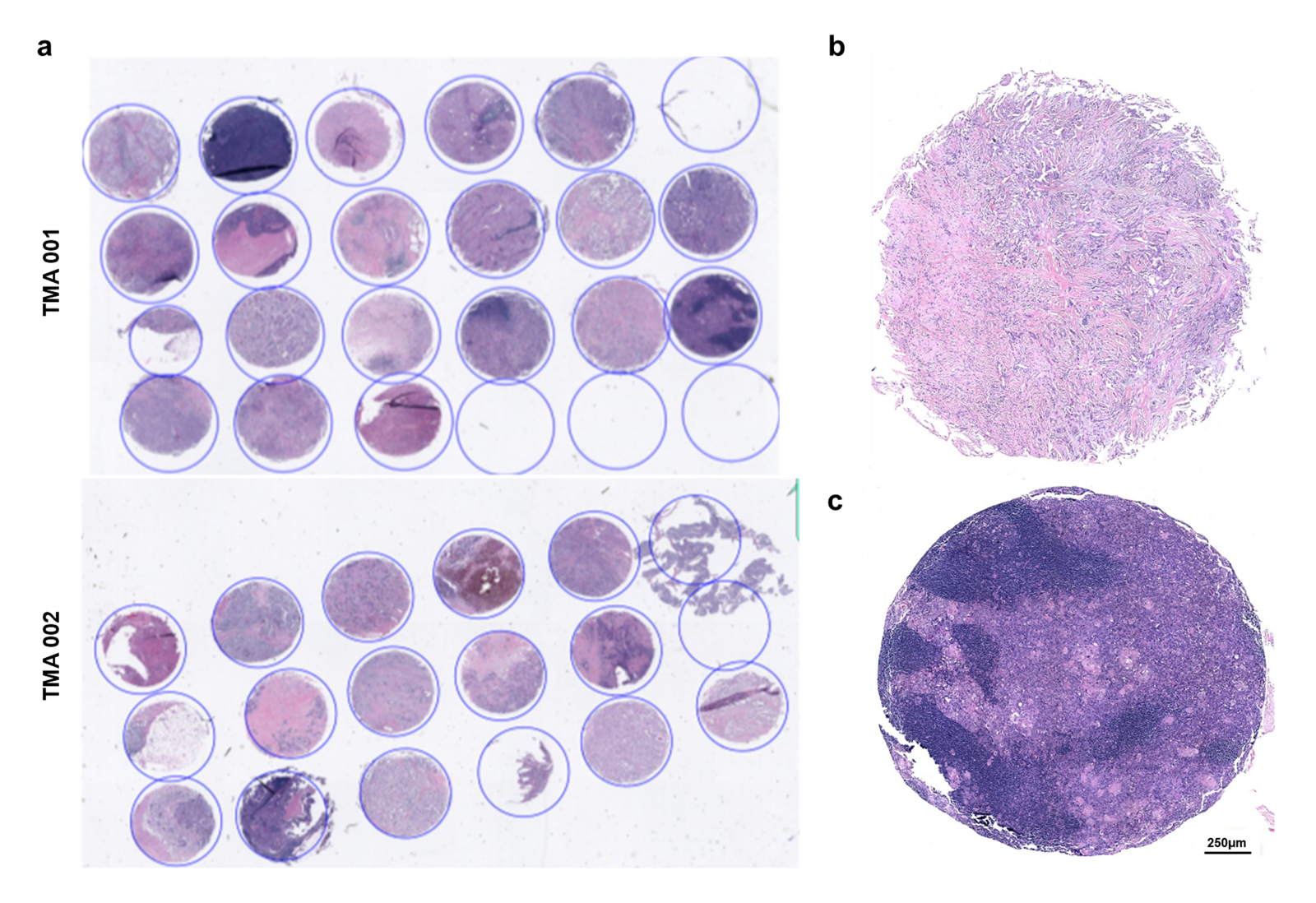


**Supp Fig 1**. **a.** Hematoxylin and Eosin staining of the TMA slides 001 and 002. Representative H&E image of **b.** Primary TNBC tissue and **c.** Matched Nodal metastasis.


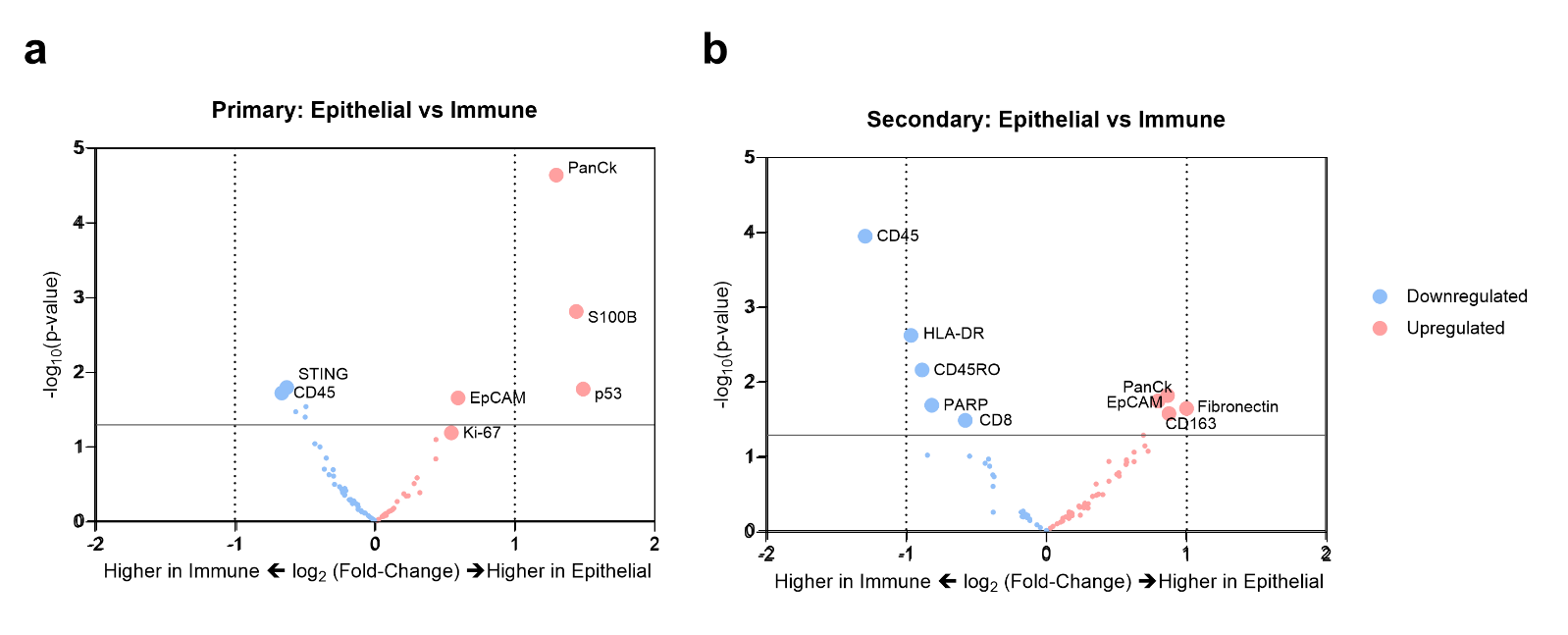


**Supp Fig 2. Microvascular signatures of epithelial vs immune regions of TNBCs in primary and secondary TNBCs.** Volcano plots of differential expression of microvasculature proteins in epithelial vs immune regions of **a.** Primary tissues and **b.** Secondary tissues. Upregulation and downregulation of proteins in secondary sites when compared to primary sites are marked in red and blue respectively. Dotted vertical lines represent log_2_(Fold-Change) of -1 and 1. Proteins above the solid horizontal line at -log_10_(p-value) = 1.3 marks p<0.05.


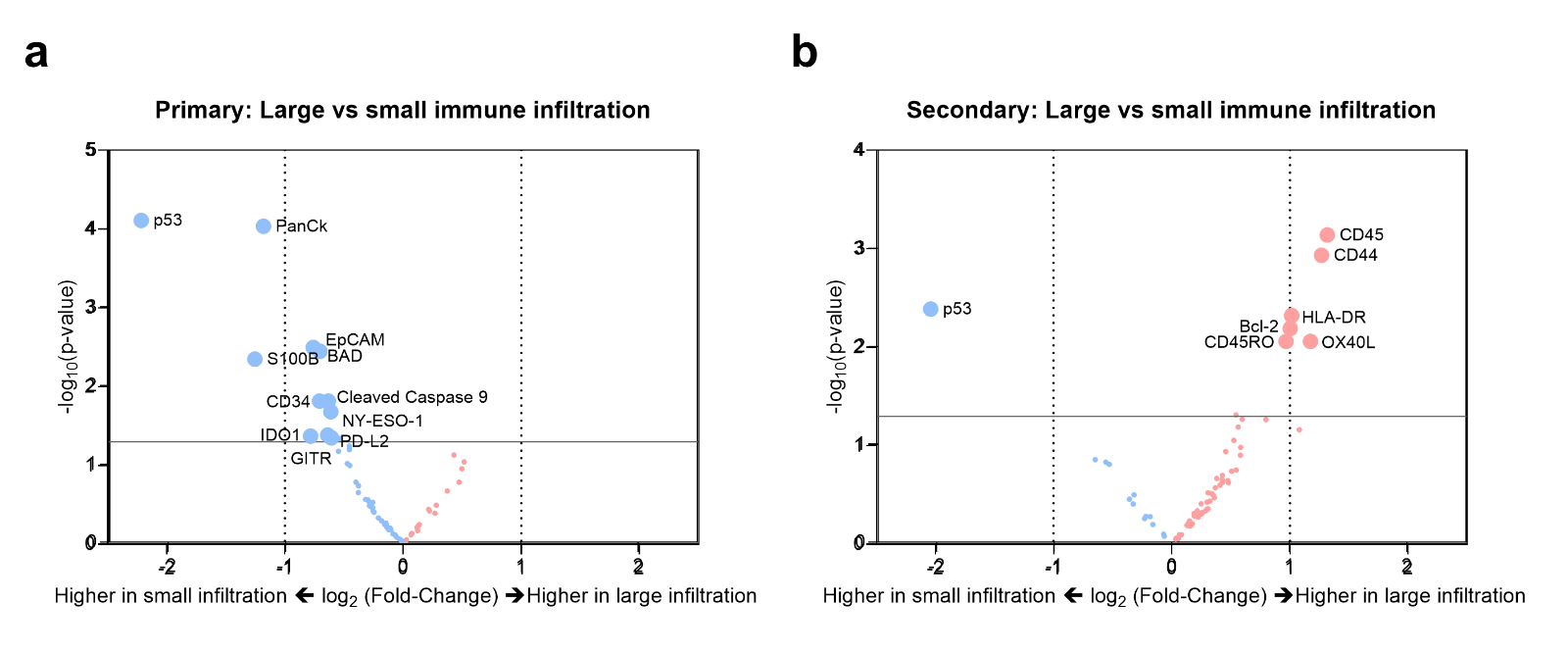


**Supp Fig 3.** **Microvasculature signatures based on size of immune infiltration (large vs small) in primary and secondary TNBCs.** Volcano plots of differential expression of microvascular proteins in large immune infiltrated sites vs small immune infiltrated sites in **a.** Primary tissues and **b.** Secondary tissue sites. Upregulation and downregulation of proteins in secondary sites when compared to primary sites are marked in red and blue respectively. Dotted vertical lines represent log_2_(Fold-Change) of -1 and 1. Proteins above the solid horizontal line at -log_10_(p-value) = 1.3 marks p<0.05.


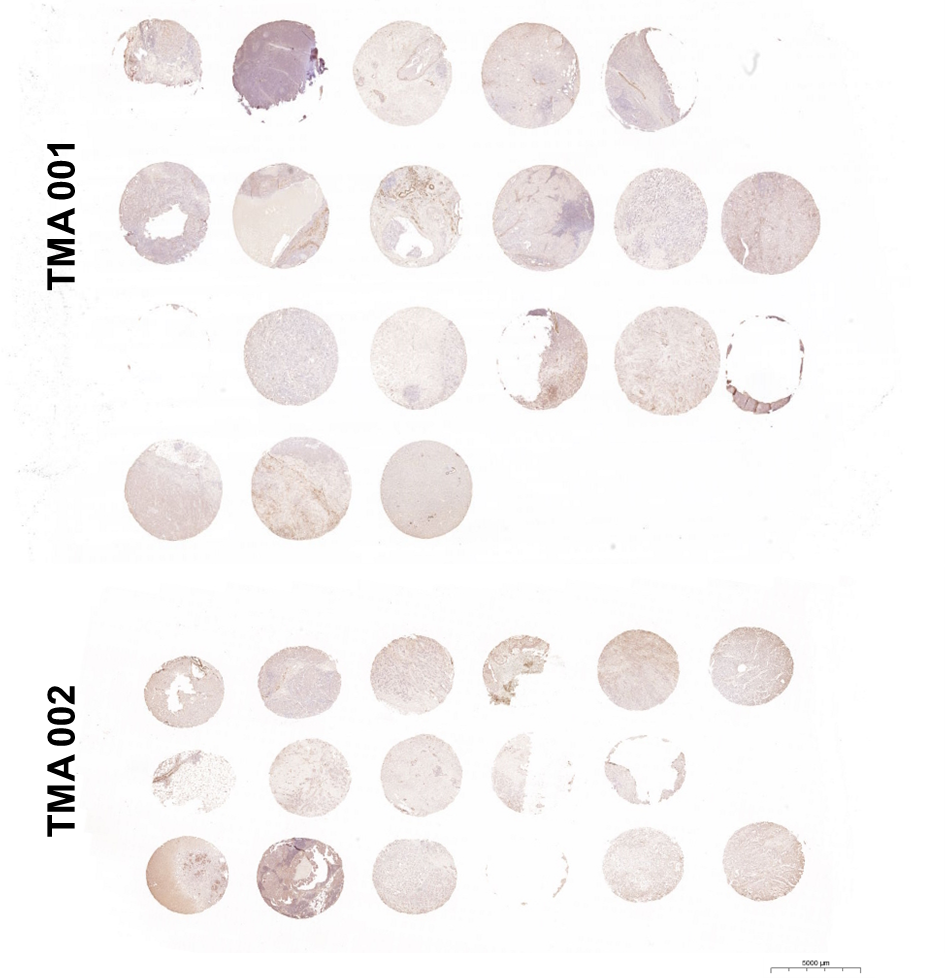


**Supp Fig 4.** **Staining for fibroblasts in the primary and secondary tissue samples on the TMAs.** Overview of full TMA scans of immunohistochemical staining for alpha Smooth Muscle Actin (aSMA) marker (scale bar: 5000µm).


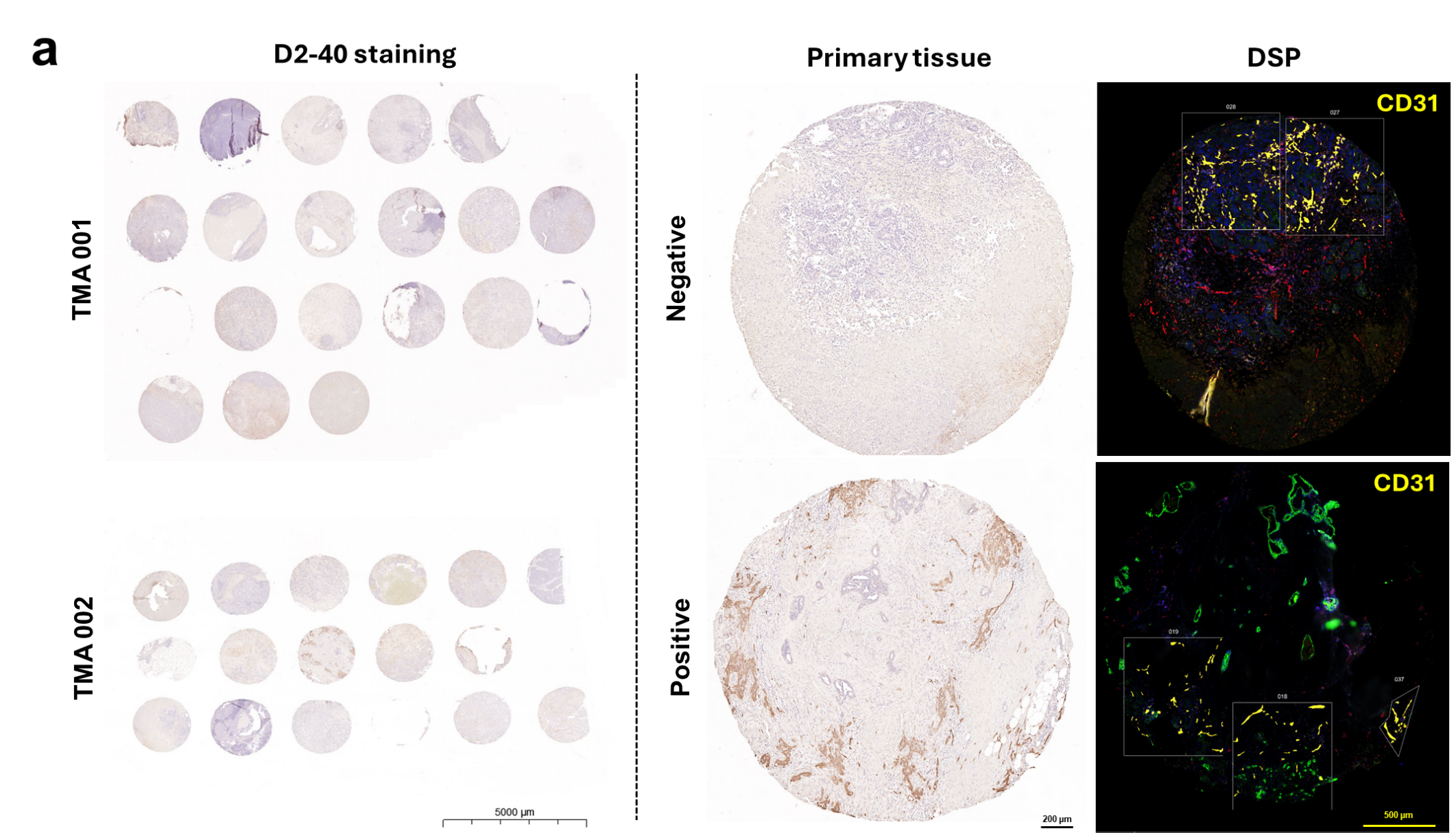


**Supp Fig 5. Staining for lymphatic vasculature in the primary and secondary tissue samples on the TMAs.** **a.** Overview of full TMA scans of immunohistochemical staining for D2-40 marker (scale bar: 5000µm). **b.** Representative images of positive and negative D2-40 staining in primary tissues (scale bar: 200µm) along with digital spatial profiling (DSP) scans showing CD31 marked areas of interest in yellow (scale bar: 500µm).
